# Comparative Study of Different Respiratory Muscle Training Methods: Effects on Cardiopulmonary Indices and Athletic Performance in Elite Short-Track Speedskaters

**DOI:** 10.3390/life14091159

**Published:** 2024-09-13

**Authors:** Tomasz Kowalski, Andrzej Klusiewicz, Kinga Rębiś, Adrian Wilk, Michał Starczewski

**Affiliations:** 1 Department of Physiology, Institute of Sport—National Research Institute, 01-982 Warsaw, Poland; 2 Department of Physical Education and Health in Biala Podlaska, Faculty in Biala Podlaska, Jozef Pilsudski University of Physical Education, 00-968 Warsaw, Poland; 3 Faculty of Rehabilitation, Jozef Pilsudski University of Physical Education, 00-968 Warsaw, Poland

**Keywords:** respiratory muscle training, exercise physiology, speedskating, voluntary isocapnic hyperpnoea, POWERbreathe, respiratory training

## Abstract

Respiratory muscle training (RMT) improves endurance performance, balance, and ability to repeat high-intensity exercise bouts, providing a rationale to be applied in short-track speedskating. To establish a preferable RMT method for short-track speedskating, the influence of inspiratory pressure threshold loading (IPTL) and voluntary isocapnic hyperpnoea (VIH) on cardiopulmonary indices and athletic performance was investigated. Sixteen elite short-track speedskaters completed 6 weeks of RMT based on IPTL or VIH. Wingate Anaerobic Tests (WAnTs), cardiopulmonary exercise tests (CPETs), spirometry assessments, and on-ice time trials were performed before and after RMT intervention. Repeated measures ANOVA was used to assess the differences between each method’s influence. No statistically significant (*p* > 0.05) differences between RMT methods were found in performance during the WAnT, CPET, or specific on-ice time trials. Spirometry measures were similar between both methods. Significant effects were found for the interaction between maximum breathing frequency during CPET (BFmax) and method (*p* = 0.009), as well as for the interaction between BFMax, method, and sex (*p* = 0.040). BFmax decreased for IPTL and increased for VIH. The interaction between method and sex revealed that BFmax increased only in males performing VIH. Our findings suggest that IPTL and VIH lead to analogous effects in the study participants, highlighting a negligible practical disparity in the impact of different RMT methods in elite short-track speedskaters.

## 1. Introduction

In recent decades, growing evidence has underscored the importance of respiratory muscle function in endurance performance [1,2,3]. However, it has become evident that traditional, sport-specific training programs may not sufficiently enhance respiratory muscle function [4,5,6]. This observation has prompted the introduction of respiratory muscle training (RMT). Remarkably, research dating back half a century already hinted at the potential to enhance the strength and endurance of respiratory muscles in healthy individuals through targeted training [7].

As presented in the recent literature, RMT improves performance in diverse scenarios, including time trials, intermittent incremental tests, and constant load tests [8,9,10]. Additionally, RMT exhibits promise in enhancing respiratory muscle endurance and strength [8,9,11], mitigating the perception of exertion or breathlessness [8], and alleviating respiratory fatigue during exercise in both normoxia and hypoxia [8,12], according to the cited studies. Furthermore, sports performance and cardiorespiratory fitness are associated with athletes’ lung function and ventilation [13]. The underlying physiological mechanisms responsible for enhancing respiratory performance entail bolstering the mechanical efficiency and fatigue resistance of respiratory muscles, thus postponing or mitigating the respiratory metaboreflex [14,15,16]. Practical implications stem from the observation that heightened fatigue and metabolite accumulation in respiratory muscles divert blood flow away from skeletal muscles, creating vasoconstriction in the exercising limbs and ultimately limiting performance. Consequently, RMT is anticipated to mitigate the respiratory metaboreflex and attenuate the associated systemic repercussions [17].

Moreover, the respiratory muscles play a crucial role in respiration and maintaining core stability [18]. When the demand for both is high, a conflict between respiratory and nonrespiratory functions may lead to impaired athletic performance, as fatigue increases faster. RMT not only strengthens the respiratory muscles but also improves their endurance. Therefore, the symptoms of fatigue associated with the high demand for respiratory muscle are delayed. Improving the coordination and integration of respiratory and stabilizing musculature during diverse sport-specific challenges such as lunging, changing direction, accelerating, and serving may enhance athletic performance [19,20] and reduce the likelihood of injury [21,22].

Furthermore, RMT leads to improved performance and exercise tolerance in repeated-sprint ability tests [23,24]. The improvement may be associated with enhanced lactate uptake and oxidative capacity, as well as improved lactate transport capacity of the trained respiratory muscles [25,26]. Respiratory muscles are sometimes described as ‘lactate netto consumers’ since increased intra-muscle lactate concentration in the absence of glycogen utilization was observed [27]. Moreover, approximately 50% of the energy used by the diaphragm comes from carbohydrate metabolism, mostly in the form of lactate utilization [28].

Finally, speedskating is one of the sports that are strongly associated with bronchial hyperresponsiveness and exercise-induced respiratory symptoms among elite athletes [29]. RMT was found to be an efficient intervention in multiple respiratory system dysfunctions and diseases [30,31]. Therefore, it may be particularly important in speedskaters as their high-intensity training routinely occurs in relatively low temperatures. Moreover, altitude training has gained more recognition in endurance sports [32], and multiple international skating arenas are situated in low or medium altitudes [33]. RMT was proven to enhance adaptation to hypoxia, improve performance at altitude, and reduce respiratory stress during altitude acclimatization [12].

This study covers two methods extensively applied in high-performance settings: inspiratory pressure threshold loading (IPTL) and voluntary isocapnic hyperpnoea (VIH). IPTL involves the use of a mechanical or electronic device that imposes resistance on inhalation. During IPTL, individuals inhale against the resistance provided by the device, which requires the respiratory muscles, particularly the diaphragm and intercostal muscles, to work harder to overcome the resistance. In most IPTL regimens, individuals are typically directed to execute complete inspirations from their residual volume while encountering resistance equivalent to over 50% of their maximal inspiratory pressure [8]. However, higher resistance up to 80% may be successfully applied in elite athletes [34]. VIH involves voluntary, rhythmic, and controlled deep breathing, typically under conditions of stable carbon dioxide levels. VIH employs equipment featuring partial rebreathing circuits and involves controlled, vigorous breathing for a duration of up to 40 min. Minimal to no external resistance is introduced, and the training stimulus is centred on deliberate hyperventilation with an intensity ranging from 60 to 90% of maximal voluntary ventilation. The goal of VIH is to challenge the respiratory system by increasing ventilation, which is accomplished by adjusting the breathing rate and depth [35].

Whereas RMT was thoroughly investigated in many endurance and team sports, its application during winter sports remains an understudied area. Considering the positive influence of RMT on endurance performance, balance, and ability to repeat high-intensity exercise bouts, we concluded that it may be particularly useful for short-track speedskaters, who have to combine speed, endurance, balance, tactical motor skills, and ability to race multiple races to achieve success during competition [36,37,38]. Therefore, we investigated the influence of IPTL and VIH on cardiopulmonary parameters and athletic performance in elite short-track speedskaters. The main objective of this study was to establish a preferred method of RMT for short-track speedskaters in the context of improved pulmonary function, inspiratory muscle strength, peak power on a cycloergometer, aerobic fitness, anaerobic capacity, and specific on-ice performance.

## 2. Materials and Methods

This study was designed as a parallel-group controlled trial and is registered at ClinicalTrials.gov under NCT05936723. This study was approved by the Institute of Sport—National Research Institute Ethics Committee (approval no KEBN-23-78-TK). All the procedures were carried out in agreement with the Declaration of Helsinki. Informed written consent was obtained from all the study participants.

### 2.1. Participants’ Characteristics

Sixteen elite short-track speedskaters took part in the study. The participants were selected through a convenience sampling method from the training group consisting of short-track speedskaters representing four distinct countries. All recruited participants held classifications within Tier 4 or Tier 5 according to the Participant Classification Framework [39], signifying their status as elite or world-class athletes. The inclusion criteria necessitated a valid medical certificate for competitive speedskating, no prior exposure to respiratory muscle training (RMT), and a minimum of six years of athletic training. The exclusion criteria encompassed any chronic medical conditions, recent acute medical conditions within the past three months, and the use of ongoing medications. The required sample size was calculated with G* Power (version 3.1.9.2; Dusseldorf, Germany) and totalled 12 participants. The following input parameters were set: effect size ƒ = 0.5, significance α = 0.05, power (1 − β) = 0.8, number of groups = 2, number of measurements = 2 (ANOVA with repeated measures, within-between interaction), in line with research in the field [11,40]. All 16 participants completed the RMT program. At the beginning of the trial, all the participants were in the base training period, having undergone 6 to 8 weeks of regular training after a post-season recovery period. As they all train under the guidance of the same coaching group, the same training program was applied. Body composition was assessed using a bioelectrical impedance analysis system (Tanita BC-420MA, Tokyo, Japan). All the measurements were taken between 7:00 and 7:30 a.m., before breakfast.

The participants were randomly assigned to either the IPTL or VIH training group. Stratified randomization was applied. First, the participants were assigned to subgroups based on their sex, and then, they were assigned to either the IPTL or VIH group based on a coin toss. Table 1 presents the participants’ baseline characteristics.

### 2.2. Respiratory Muscle Training Protocols

**Figure 1:**
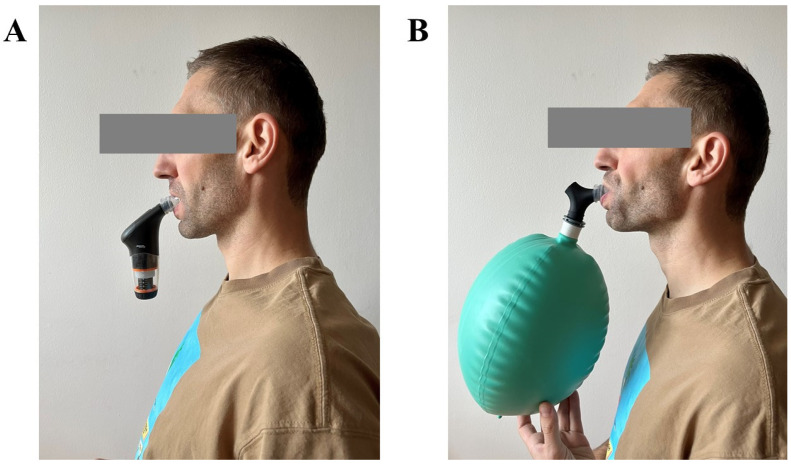
Illustrative application of two RMT methods. (**A**) Presentation of inspiratory pressure threshold loading. (**B**) Presentation of voluntary isocapnic hyperpnoea.

The participants completed the 6-week RMT program, assigned to either the IPTL or VIH training group (see Figure 1). The direct training supervision was limited to the first week and last three weeks of the intervention, as the training was performed during the group training camps. The participants were regularly reminded by the coaching team about following the RMT program.

IPTL group trained 5 days a week, two times per day, with at least a 6 h break between sessions. Each session consisted of 30 dynamic inspiratory manoeuvres. The participants were instructed to perform full vital capacity breaths from the residual volume level, against a resistance allowing them to perform 28–34 powerful and dynamic inspirations. The participants were instructed to increase the resistance periodically to account for training improvement. POWERbreathe Plus—Medium Resistance devices (POWERbreathe International Ltd., Southam, UK) were used.

The VIH group trained every second day, with gradual progression based on breathing frequency and session length. The first session was 3 min of exercise with a frequency of 20 breaths·min^−1^, and either 1 min or 2 breaths·min^−1^ were added with each consecutive session. Isocapnic BreathWayBetter devices (Isocapnic Technologies Inc., Kelowna, BC, Canada) with 6 L bags were used. The VIH training program is presented in Table 2.

### 2.3. Data Collection

The technical team performing the tests and measurements was blinded to the type of intervention. The data were collected in the same way for both the IPTL and VIH groups, applying the following procedures.

Wingate Anaerobic Tests (WAnTs) were conducted using the Monark 874E Cycle Ergometer (Monark Exercise AB, Vansbro, Sweden). Before the WAnTs, a warm-up of 5 min with resistance ranging from 0.8 to 1.2 W/kg was carried out. Subsequently, participants engaged in a maximal 6 s sprint, with the resistance adjusted to 7.5% of their individual body mass. After a 2 min rest interval, the athletes underwent a 30 s WAnT with resistance set at 7.5% of the individual body mass. The primary goal for the participants was to attain the highest peak power output as quickly as possible and maintain the highest power output throughout the entire duration of the test. The athletes received enthusiastic and motivating verbal support. Maximum power output (PmaxWanT) and total work (TW-AnC), reflecting anaerobic capacity, were measured. Both parameters were computed using dedicated software (MCE 6.0, JBA Z. Staniak, Warsaw, Poland) connected to the cycle ergometer. Blood samples were taken from their fingertips 3′ after the cessation of the exercise and added into 20 uL capillary tubes to determine the peak blood lactate concentration (bLaWAnT). The assays were performed with a Super GL2 analyzer (Dr. Müller Gerätebau GmbH, Freital, Germany). WAnTs took place between 9:30 and 10:30 a.m.

Cardiopulmonary exercise testing (CPET) was performed with the Cortex Metamax B3 (Cortex Biophysik GmbH, Leipzig, Germany), breath-by-breath method, and a Cyclus II Ergometer (RBM, Leipzig, Germany). Participants underwent an incremental ramp test until exhaustion, commencing at 55–70 W and incrementally increasing the load by 0.17–0.28 W·s^−1^, individually adjusted for body mass. Maximum heart rate (HRmax), oxygen uptake (VO_2_max), respiratory exchange ratio (RERmax), ventilation (VEmax), breathing frequency (BFmax), tidal volume (TVmax), and power (PmaxCPET) were established. Moreover, maximum blood lactate concentration (bLaMaxCPET) was measured. Blood samples were taken from the fingertip immediately after and 3′ after the cessation of the exercise into 20 μL capillary tubes. The highest value was used in a further analysis, denoted as bLaMaxCPET. The assays were performed with a Super GL2 analyzer (Dr. Müller Gerätebau GmbH, Freital, Germany). All the maximum cardiopulmonary indices were the highest averages over 15 s. Ventilatory thresholds were determined with Cortex Metasoft™ (Cortex Biophysik GmbH, Leipzig, Germany) and the V-slope method, with visual correction when necessary [41]. A standard correction of 15 s, based on the mean response time representing the cardiopulmonary response lag in metabolic demand in a slow ramp test, was applied [42]. Heart rate, ventilation, breathing frequency, tidal volume, and power values corresponding to Ventilatory Threshold 1 and Ventilatory Threshold 2 were established (VT1-HR, VT1-VE, VT1-BF, VT1-TV, VT1-P, VT2-HR, VT2-VE, VT2-BF, VT2-TV, and VT2-P). CPET took place on the same day, at least 3 h after the WAnTs.

Contec SP80B (Contec Medical Systems, Qinhuangdao, China) was used to perform the spirometry assessment. European Respiratory Society guidelines for conducting spirometry were applied [43]. Forced vital capacity (FVC), forced expiratory volume in 1 s (FEV1), forced expiratory volume in 1 s to forced vital capacity ratio (FEV1/FVC), and peak expiratory flow (PEF) were measured. Inspiratory muscle strength was assessed using the S-Index Test, conducted with the POWERbreathe K5 device (POWERbreathe International Ltd., Southam, UK). The test involved eight forceful and dynamic inspiratory manoeuvres from residual volume to full inspiratory capacity, performed while standing after a warm-up consisting of 10 inspiratory manoeuvres, according to the guidelines for athletic settings [44]. The highest achieved value from a single manoeuvre was recorded as the S-Index Test score. All the spirometry measurements were taken on the next day after the WAnTs and CPETs, between 7:30 and 8:30 a.m.

Time measurements were taken to assess specific on-ice performance. Fastest lap time (FLT) was measured as an individual time trial with maximum effort for one ice-rink lap, corresponding to 111.12 m. The athletes’ goal was to achieve the highest speed available for one lap. The trial was performed with a flying start and 1.5 ice-rink laps of acceleration before the timed lap. The relay simulation was performed with 8 rounds of 2 maximum speed ice-rink laps (16.60–19.74 s) with the active recovery of easy skating (105.60–118.44 s). The session was designed to mimic the competition demands of short-track 3000 m relay, as performed in international events, and allow for an effective group training session. The recovery slightly varied and was adjusted to other athletes’ performance, as they completed their maximum speed laps during this time. This was the standard training design, and all the participants were familiar with the protocol. The athletes’ goal was to achieve the highest average speed in the maximum speed laps across the whole session. Out of 16 maximum speed laps covered by each athlete, the fastest and the slowest laps were rejected to establish the average lap time for the remaining 14 laps (RS-ALT). All the time measurements were performed by the coaching team with Seiko S141 (Seiko Watch Corporation, Tokyo, Japan) stopwatches. All the trials were performed in repeatable conditions, one day after performing the WAnTs and CPETs, between 10:00 and 12:00 a.m.

Some athletes dropped out from the selected testing procedures due to health-related issues: 2 athletes did not complete the post-RMT WAnT, 1 athlete did not complete the post-RMT CPET, and 1 athlete did not complete the post-RMT time trials on ice. Despite this, the required sample size was met.

### 2.4. Statistical Analysis

The assessment of differences in participants’ baseline characteristics between the groups was performed with independent *t*-tests. The normality of the distribution was assessed with the Shapiro–Wilk test and a visual analysis of the plot figures. The main within-between effects for the interaction between time and training method, and the interaction between time, training method, and sex were assessed by repeated-measures analysis of variance (ANOVA). Additionally, homogeneity was assessed with Levene’s test. In significant main effects, a post hoc assessment was performed using Bonferroni correction. Significance was set at *p* < 0.05. The results are presented as means and standard deviations. Partial-eta squared (pη2) and omega squared (ω^2^) were used to determine the effect size. All statistical analyses were performed using the JASP Team statistical package JASP (Amsterdam, The Netherlands, Version 0.17.2).

## 3. Results

The values of pulmonary function, inspiratory muscle strength, peak power on a cycloergometer, aerobic fitness, anaerobic capacity, and specific on-ice performance before and after the intervention are presented in Table 3, Table 4, Table 5, Table 6 and Table 7.

There was a statistically significant effect for BFmax and method (F(1, 2) = 9.892, *p* = 0.009, η_p_^2^ = 0.306, ω^2^ = 0.026). Moreover, there was a statistically significant effect for method and sex interaction (F(1, 2) = 5.398, *p* = 0.040, η_p_^2^ = 0.329, ω^2^ = 0.030). The mean BFmax decreased for IPTL and increased for VIH. However, a significant effect for method and sex interaction revealed that BFmax increased only in males performing VIH. There were no other significant effects for any monitored parameters.

## 4. Discussion

The main goal of this study was to assess two RMT methods and their influence on elite speedskaters. To our knowledge, this is one of the first studies regarding RMT in winter sports [45]. We investigated the effects of RMT in relation to a wide range of spirometry indices, inspiratory muscle strength, aerobic fitness, peak power, and anaerobic capacity on a cycloergometer, as well as specific on-ice performance. No statistically significant differences between methods were found in athletic performance, spirometry measures, and inspiratory muscle strength. Ventilatory thresholds obtained during CPET do not significantly differ between methods for heart rate, power output, ventilation, and breathing frequency. Only significant effects for BFmax and method (*p* = 0.009), as well as for the interaction between BFMax, method, and sex (*p* = 0.040) were found. Our findings suggest that both RMT methods elicited similar changes in study participants and that there is a very limited practical difference between the influence of IPTL and VIH on elite short-track speedskaters.

Our findings coincide with the results of a definitive review and meta-analysis from Illi et al. (2012), suggesting that the practical differences between the influence of IPTL and VIH are not significant [9]. Klusiewicz et al. (2019) also report similar changes induced by different RMT methods; however, both methods were ITPL-based, and only the load progression was different [45]. More recent studies also reported similar effects of different RMT methods on respiratory and athletic performance in active young males [40]. Although the difference in BFmax between the methods was observed, it is not clinically or practically significant. Noteworthy, the conclusions should not be applied to people with disabilities or patients, as multiple studies suggest that IPTL is a more efficient RMT method in the aforementioned populations [46,47]. Moreover, another exemplary review from HajGhanbari et al. (2013), suggests that noteworthy differences between the training effects of both methods do exist due to training method specificity [8]. As IPTL is more strength-oriented, it results in larger improvements in maximal inspiratory pressures. As VIH is more endurance-oriented, it is associated with higher flow rates, improved maximal voluntary ventilation, and larger velocities of respiratory muscle contraction.

Therefore, two important factors are worth considering when discussing RMT methods: individual needs of an athlete and performance determinants. Speculatively, athletes with low endurance and high levels of inspiratory muscle strength probably benefit more from VIH, and athletes with excellent endurance and low levels of inspiratory muscle strength should consider IPTL as their first choice. Athletic performance determinants are widely applied to optimize training processes and predict performance, among others [48]. However, research specific to breathing patterns, spirometry measures, and respiratory muscle function remains scarce and is rarely included in athletic performance models. Although respiratory function and performance were investigated in many sports [3,34,49,50], no clear guidelines on optimal inspiratory muscle strength levels or spirometry measures were presented. The lack of evidence-based data regarding the most appropriate values for specific athletic performance presents a knowledge gap for researchers and requires coaches to design RMT based on their personal experience and intuition.

Noteworthily, the study participants noted both positive and negative adaptations in monitored parameters. Despite associating the direction and magnitude of changes with experimental interventions in many studies, we do not link RMT with observed pre- and post-intervention differences. RMT constitutes only a small percentage of the training load, i.e., analogous RMT programs amounted to only 0.83–6.61% of the total training load in well-trained athletes [51]. Therefore, the rest of the training program probably has an adequately larger influence on the monitored parameters, as corresponding with the remaining training load. Our study design allows for an upright comparison of the two most popular RMT methods without considering variables like the effectiveness of the overall training program, psychophysiological fatigue, or adequacy of total training load. Therefore, we do not associate the mixed outcome of the interventions with their design, as interventions similar in training method and duration have been proven effective in the literature [3,52,53]. It is possible, however, that different RMT programmes might be more effective in elite short-track speedskaters. Not all previous studies showed improvement associated with RMT, and the application of specific and individually tailored RMT protocols have been recommended [54].

Although RMT was investigated in multiple sports and settings, many areas remain underrepresented. Winter sports generate less scientific interest compared to summer sports [55]. Consequently, the application of RMT in winter sports requires further investigation in many areas. Moreover, optimal levels of inspiratory muscle strength levels or spirometry measures still need to be established in the context of sport-specific performance. Finally, studies on short-track speedskaters are rare, and only limited research on speedskating training and performance is available.

## 5. Strengths and Limitations

This study investigated a homogeneous group of elite short-track speedskaters, which may be considered as a study strength. However, considering the unique characteristics of elite athletes, the findings should not be extrapolated to different populations. Large female participation and the application of a wide array of performance measurements are noteworthy aspects of the study. It is worth mentioning that the technical team performing the tests and measurements was blinded to the type of intervention. The lack of a sham control group and direct training supervision during the whole duration of the intervention may be considered a study limitation. Although the sample size requirements were met, the number of participants was small.

## 6. Conclusions

The primary objective of the study was to evaluate the influence of two RMT methods on elite short-track speedskaters. The examination involved an array of aspects, including pulmonary function, inspiratory muscle strength, aerobic endurance, peak power, and anaerobic capacity tested on a cycloergometer, in addition to the skaters’ performance on the ice. Our findings suggest that IPTL and VIH lead to analogous effects in the study participants, highlighting a negligible practical disparity in the impact of different RMT methods in elite short-track speedskaters.

## Figures and Tables

**Table 1:** Baseline participant characteristics.

Variable/Group	IPTL (*n* = 8)	VIH (*n* = 8)
Age (years)	23.06 ± 2.36	22.18 ± 2.64
Body mass (kg)	67.96 ± 8.78	69.0 ± 9.70
Body height (cm)	172.88 ± 6.49	175.12 ± 7.90
Body fat (%)	11.15 ± 4.43	13.66 ± 5.49
VO_2_max (mL·min^−1^·kg^−1^)	56.88 ± 6.36	53.13 ± 7.64
Training experience (years)	11.92 ± 3.06	12.1 ± 3.43

Values are mean ± standard deviation. IPTL, inspiratory pressure threshold loading, VIH, voluntary isocapnic hyperpnoea, VO_2_max—maximum oxygen uptake. No statistically significant differences in any characteristic were found between the groups (*p* > 0.05). In each group, there were 3 (37.5%) females and 5 (62.5%) males.

**Table 2:** The voluntary isocapnic hyperpnoea (VIH) group 6-week training program.

Session Number	Session Length (Minutes)	Breathing Frequency (Breaths per Minute)	Session Number	Session Length (Minutes)	Breathing Frequency (Breaths per Minute)
1	3	20	12	13	22
2	4	20	13	14	24
3	5	20	14	15	24
4	5	20	15	16	24
5	6	22	16	17	24
6	7	22	17	18	44
7	8	22	18	18	26
8	9	22	19	19	26
9	10	22	20	20	26
10	11	22	21	20	26
11	12	22			

**Table 3:** Maximum parameters obtained during WAnT before and after 6-week respiratory muscle training.

Variables/Group	IPTL (*n* = 7)		VIH (*n* = 7)		*p*-Value
	Before	After	Before	After	
PmaxWAnT (W·kg^−1^)	13.56 ± 1.70	13.04 ± 1.56	12.98 ± 1.23	13.11 ± 0.81	0.068
TW-AnC (kJ·kg^−1^)	4.68 ± 0.48	4.57 ± 0.34	4.41 ± 0.45	4.52 ± 0.24	0.101
bLaWAnT (mmol·L^−1^)	16.59 ± 2.73	13.30 ± 2.10	15.94 ± 1.60	14.88 ± 1.12	0.861

The values are means ± standard deviations. *p*-value refers to the effects for the interaction between time and training method. Abbreviations: IPTL—inspiratory pressure threshold loading, VIH—voluntary isocapnic hyperpnoea, PmaxWanT—maximum power output, TW-AnC—total work, bLaWAnT—peak blood lactate concentration.

**Table 4:** Maximum parameters obtained during cardiopulmonary exercise testing before and after 6-week respiratory muscle training.

Variables/Group	IPTL (*n* = 7)		VIH (*n* = 8)		*p*-Value
	Before	After	Before	After	
PmaxCPET (W·kg^−1^)	4.82 ± 0.59	4.78 ± 0.62	4.47 ± 0.61	4.03 ± 1.68	0.302
VO_2_max (mL·min^−1^·kg^−1^)	56.88 ± 6.34	56.375 ± 7.50	54.43 ± 7.23	54.71 ± 5.91	0.510
HRmax (bpm)	196.62 ± 8.23	191.00 ± 13.75	192.25 ± 6.84	188.43 ± 6.58	0.560
VEmax (L·min^−1^)	162.82 ± 33.73	157.60 ± 32.41	153.77 ± 39.50	164.27 ± 31.53	0.274
BFmax (brpm)	61.13 ± 6.20	55.00 ± 5.93	55.43 ± 6.13	58.43 ± 8.67	0.009
TVmax	2.88 ± 0.47	2.86 ± 0.48	2.80 ± 0.77	2.92 ± 0.69	0.965
RERmax	1.15 ± 0.04	1.18 ± 0.05	1.15 ± 0.04	1.21 ± 0.03	0.318
bLaMaxCPET (mmol·L^−1^)	14.30 ± 2.87	11.92 ± 2.40	12.99 ± 3.69	13.20 ± 2.29	0.180

The values are means ± standard deviations. *p*-value refers to the effects for the interaction between time and training method. Abbreviations: IPTL—inspiratory pressure threshold loading, VIH—voluntary isocapnic hyperpnoea, PmaxCPET—maximum power output, VO_2_max—maximum oxygen uptakeHRmax—maximum heart rate, VEmax—maximum ventilation, BFmax—maximum breathing frequency, TVmax—maximum tidal volume, RERmax—maximum respiratory exchange ratio, bLaMaxCPET—maximum blood lactate concentration, bpm—beats per minute, brpm—breath rate per minute.

**Table 5:** Spirometry assessment and inspiratory muscle strength before and after 6-week respiratory muscle training.

Variables/Group	IPTL (*n* = 8)		VIH (*n* = 8)		*p*-Value
	Before	After	Before	After	
FVC (L)	5.61 ± 1.06	5.38 ± 0.84	5.31 ± 1.03	5.21 ± 1.06	0.604
FEV1 (L)	4.44 ± 0.71	4.42 ± 0.65	4.65 ± 1.03	4.54 ± 1.06	0.591
FEV1/FVC	79.98 ± 9.61	82.68 ± 6.26	87.14 ± 6.55	86.76 ± 8.87	0.326
PEF (L·min^−1^)	6.62 ± 2.09	7.97 ± 2.71	8.77 ± 2.93	8.88 ± 3.22	0.323
S-Index Test Score (cmH_2_O)	142.39 ± 26.96	141.64 ± 22.84	137.83 ± 29.57	152.00 ± 29.53	0.065

The values are means ± standard deviations. *p*-value refers to the effects for the interaction between time and training method. Abbreviations: IPTL—inspiratory pressure threshold loading, VIH—voluntary isocapnic hyperpnoea, FVC—forced vital capacity, FEV1—forced expiratory volume in 1 s, FEV1/FVC—forced expiratory volume in 1 s to forced vital capacity ratio, PEF—peak expiratory flow.

**Table 6:** Ventilatory thresholds before and after 6-week respiratory muscle training.

Variables/Group	IPTL (*n* = 7)		VIH (*n* = 8)		*p*-Value
	Before	After	Before	After	
VT1-HR (bpm)	163.50 ± 13.89	164.38 ± 17.57	153.00 ± 11.55	156.43 ± 4.83	0.455
VT1-P (W·kg^−1^)	2.81 ± 0.77	3.06 ± 0.68	2.52 ± 1.04	2.85 ± 0.65	0.564
VT1-VE (L·min^−1^)	63.51 ± 16.95	73.575 ± 14.69	59.94 ± 22.04	67.70 ± 14.94	0.876
VT1-BF (brpm)	29.75 ± 6.089	32.38 ± 5.50	27.75 ± 4.39	30.09 ± 5.08	0.618
VT1-TV (L)	2.13 ± 0.29	2.28 ± 0.39	2.16 ± 0.65	2.25 ± 0.46	0.703
VT2-HR (bpm)	182.66 ± 10.34	178.63 ± 16.13	174.75 ± 12.209	174.57 ± 5.41	0.263
VT2-P (W·kg^−1^)	3.90 ± 0.83	4.50 ± 0.85	3.36 ± 1.06	3.69 ± 0.77	0.124
VT2-VE (L·min^−1^)	92.93 ± 17.85	98.76 ± 16.95	85.80 ± 25.79	101.53 ± 18.40	0.579
VT2-BF (brpm)	38.13 ± 5.64	39.50 ± 4.18	35.75 ± 4.95	40.71 ± 4.99	0.216
VT2-TV (L)	2.44 ± 0.38	2.51 ± 0.36	2.41 ± 0.76	2.49 ± 0.60	0.945

The values are means ± standard deviations. *p*-value refers to the effects for the interaction between time and training method. Abbreviations: IPTL—inspiratory pressure threshold loading, VIH—voluntary isocapnic hyperpnoea, VT1-HR—heart rate at Ventilatory Threshold 1, VT1-P—power at Ventilatory Threshold 1, VT1-VE—ventilation at Ventilatory Threshold 1, VT1-BF—breathing frequency at Ventilatory Threshold 1, VT1-TV—tidal volume at Ventilatory Threshold 1, VT2-HR—heart rate at Ventilatory Threshold 2, VT2-P—power at Ventilatory Threshold 2, VT2-VE—ventilation at Ventilatory Threshold 2, VT2-BF—breathing frequency at Ventilatory Threshold 2, VT2-TV—tidal volume at Ventilatory Threshold 2, bpm—beats per minute, brpm—breath rate per minute.

**Table 7:** On-ice performance results before and after 6-week respiratory muscle training.

Variables/Group	IPTL (*n* = 7)		VIH (*n* = 8)		*p*-Value
	Before	After	Before	After	
FLT (s/lap)	8.83 ± 0.47	8.62 ± 0.22	9.03 ± 0.68	8.70 ± 0.35	0.394
RS-ALT (s/lap)	9.27 ± 0.09	9.21 ± 0.27	9.49 ± 0.32	9.29 ± 0.33	0.334

The values are means ± standard deviations. *p*-value refers to the effects for the interaction between time and training method. Abbreviations: IPTL—inspiratory pressure threshold loading, VIH—voluntary isocapnic hyperpnoea, FLT—fastest lap time, RS-ALT—relay simulation—average lap time.

## Data Availability

Data will be made available upon reasonable request to the corresponding author (T.K.).
